# Conformational Screening of Arbidol Solvates: Investigation via 2D NOESY

**DOI:** 10.3390/pharmaceutics15010226

**Published:** 2023-01-09

**Authors:** Varvara A. Eventova, Konstantin V. Belov, Sergey V. Efimov, Ilya A. Khodov

**Affiliations:** 1 Krestov Institute of Solution Chemistry, Russian Academy of Sciences, Ivanovo 153045, Russia; 2 Department of Inorganic Chemistry, Ivanovo State University of Chemistry and Technology, Ivanovo 153000, Russia; 3 Institute of Physics, Kazan Federal University, Kazan 420008, Russia

**Keywords:** Arbidol, small molecules, spatial structure, NMR, NOESY, solvatomorphism, polymorphism

## Abstract

Understanding of the nucleation process’s fundamental principles in saturated solutions is an urgent task. To do this task, it is necessary to control the formation of polymorphic forms of biologically active compounds. In certain cases, a compound can exist in a single polymorphic form, but have several solvates which can appear in different crystal forms, depending on the medium and conditions of formation, and show different pharmaceutical activity. In the present paper, we report on the analysis of Arbidol conformational preferences in two solvents of different polarities—deuterated chloroform and dimethyl sulfoxide—at 25 °C, using the 2D NOESY method. The Arbidol molecule has various solvate forms depending on the molecular conformation. The method based on the nuclear Overhauser effect spectroscopy was shown to be efficient in the analysis of complex heterocyclic compounds possessing conformation-dependent pseudo-polymorphism. It is one of the types of polymorphism observed in compounds forming crystal solvates. Combined use of NMR methods and X-ray data allowed determining of conformer populations of Arbidol in CDCl_3_ and DMSO-d_6_ which were found to be 8/92% and 37/63%, respectively. The preferred conformation in solution is the same that appears in stable crystal solvates of Arbidol.

## 1. Introduction

Arbidol^®^ (ethyl 6-bromo-4-[(dimethylamino)methyl]-5-hydroxy-1-methyl-2- (phenylsulfanylmethyl)indole-3 carboxylate), in the form of hydrochloride monohydrate, is an immunomodulating drug which has been widely used in Russia and China [1]. Arbidol^®^ (also called umifenovir) hydrochloride monohydrate is used for preventing and treating acute respiratory infections, including influenza [2], and for inhibiting the infection caused by SARS-CoV-2 [3,4]. Functional groups of umifenovir—hydroxy, amino and carboxy—can interact giving rise to different synthones with hydrogen bonds. Antiviral activity and allowable cytotoxicity profiles [3,5] make it a promising candidate for further investigations as a potential therapeutic agent in selective treatment of flavivirus infections [6]. Spatial structure of umifenovir thus plays an important role in improving the existing and creating new drug compounds.

Umifenovir in solid phase exists as a large number of crystal solvates that appear in various crystal forms, depending on the medium and synthesis conditions; at the same time, there is only one polymorphic form. As well as having a number of active pharmaceutical ingredients (APIs) studied so far on the example of imendazole derivatives [7,8], it demonstrates conformation-dependent pseudo-polymorphism (the particular case of polymorphism met in solvate crystal compounds [9,10,11,12,13]). Thus, correlation between the conformational state of the API molecule in the saturated solution and the molecular structure in the solid phase allows performing fast screening [14] and determining the probability of obtaining a certain solvate form, rather than the polymorphic form. Studies on this topic, however, are often based solely on X-ray structural analysis [15,16]. Therefore, it is necessary to develop approaches capable of preliminary estimating of the producing probability of a desired API form on early stages of the mechanochemical synthesis. Nuclear Overhauser effect spectroscopy (NOESY) was shown to be an efficient method of finding small molecules conformers in solutions [11,17,18,19,20,21] and in fluids [22,23,24,25], in the case of conformation-dependent polymorphism of drug compounds, which is the factor defining the obtained solid phase. This approach can be effective, both for understanding the processes occurring in the pre-nucleation state and for predicting the probability of obtaining certain polymorphic forms.

To check this idea, geometry characteristics obtained in NOESY experiments were compared directly with the results of the X-ray analysis. Conformers of umifenovir given by X-ray were combined in conformer groups for both the polymorph (P) and for the solvates (S). To predict the most probable conformers, distances between atoms in umifenovir dissolved in CDCl_3_ and DMSO-d_6_ obtained by NOESY and configurations given by X-ray diffractometry were used. The obtained results turned out to be quite surprising.

## 2. Materials and Methods

Samples of umifenovir (declared purity ≥98% (HPLC) CAS no. 131707-23-8), CDCl_3_ (99.8 atom % D; CAS no. 865-49-6) and anhydrous DMSO-d_6_ (99.9 atom % D; CAS no. 2206-27-1) were purchased from “Sigma-Aldrich Rus” and used without further purification. A saturated solution of umifenovir in chloroform was prepared in a standard 5-mm NMR tube by adding the compound into the solvent until the stable solid phase appeared; 20 mg of the compound had been added finally into 1 mL of the solvent.

NMR spectra were recorded on a Bruker Avance III 500 spectrometer, equipped with a 5-mm probe head and using TopSpin 3.6.1 software. The temperature value of 25 °C was controlled by chilled air flow using Bruker BVT-2000 and BCU-05 units. Assignment of cross-peaks in the NOESY spectra was achieved using 1D (^1^H, ^13^C; see Figures S1 and S2) and 2D (^1^H-^13^C HSQC, ^1^H-^13^C HMBC, ^1^H-^1^H TOCSY; Figures S3–S7) experiments, literature data and chemical shift tables from AIST (Spectral Database for Organic Compounds). Integral intensities of the signals were obtained from the NOESY spectra gained using the NOESY standard pulse program. Spectral width was set to 12 ppm in both dimensions; number of scans was 64; relaxation delay was 4.5 s; mixing time varied from 0.4 to 0.6 s in steps of 0.05 s. Obtained information on the chemical shifts in 1D spectra and on intramolecular interactions of types H–H and H–C is presented in Table S1.

Note that the information on the umifenovir conformers’ structure, necessary for interpretation of the NMR spectra, was provided by X-ray structural analysis [26] (see Table S2).

## 3. Results and Discussion

**Figure 1:**
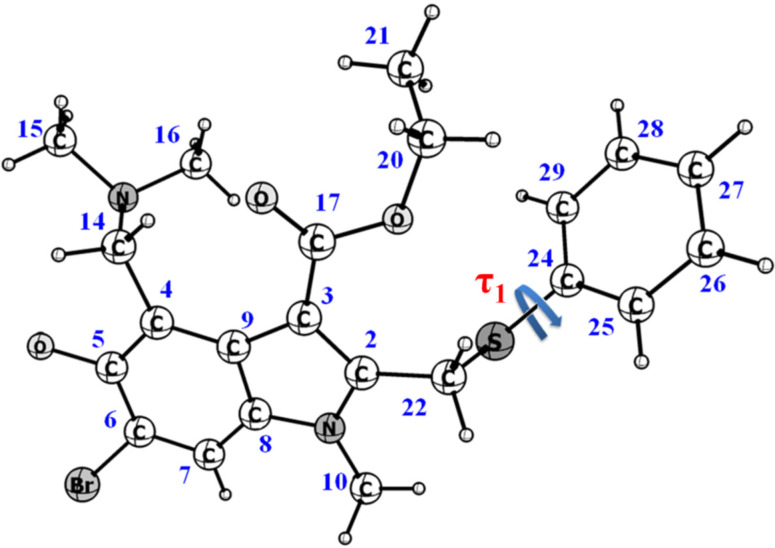
Chemical structure of umifenovir (Arbidol), showing the dihedral angle responsible for appearing of conformers.

According to the Cambridge Crystallographic Data Centre [https://www.ccdc.cam.ac.uk/ (accessed 31 October 2022)], conformations of umifenovir molecules comprising the polymorph and solvates are characterized by the dihedral angle τ_1_ (C_22_–S_1_–C_24_–C_29_) (see Figure 1 and Table S3). Depending on the angle τ_1_ and the orientation of the phenyl group, with respect to the indole moiety, six possible conformers can be observed [27,28]; they can be divided into two groups, based on the angle values: S having τ_1_ about −100° ± 20 and P with τ_1_ ≈ 170°.

**Figure 2:**
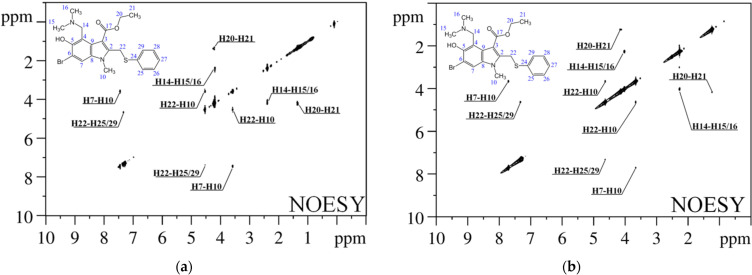
^1^H–^1^H NOESY spectra of umifenovir in CDCl_3_ (**a**) and DMSO-d_6_ (**b**) (500 MHz).

The peculiarity of the present study is that the conformer fractions in CDCl_3_ and DMSO-d_6_ were calculated based on the experimental interatomic distances obtained in X-ray studies, assumed to be the most probable in condensed matter. Distances in the dissolved molecules were determined from experimental cross-relaxation rates, obtained by analysis of the NOESY spectra (Figure 2). Five pairs of cross-peaks in the NOESY spectrum correspond to intramolecular distance within 5 Å (H7–H10, H10–H22, H14–H15/H16, H20–H21 and H22–H25/H29).

Distances corresponding to the observed cross-peaks were determined for each conformer according to the distance averaging models (Equations (1) and (2)) suitable for different atomic groups, allowing for their intramolecular mobility [18,19,29,30]:

(1) $${r_{i}}^{eff}={\left[\frac{1}{n_{I}n_{S}}\sum_{i}\frac{1}{r_{i}^{6}}\right]}^{-\frac{1}{6}}$$

(2) $${r_{i}}^{eff}={\left[\frac{1}{5}\sum_{k=-2}^{2}{\left|\frac{1}{3}\sum_{i=1}^{3}\frac{Y_{2k}(\theta_{mol}^{i},\varphi_{mol}^{i})}{r_{i}^{3}}\right|}^{2}\right]}^{-\frac{1}{6}}$$

Here *θ^i^_mol_* and *φ^i^_mol_* are the polar angles of the internuclear vector in the molecular reference frame and *Y*_2*k*_ are the second-order spherical harmonics with the coefficients proposed in [18]. Distance H7–H10 was chosen as the reference interatomic distance, and H22–H25/H29 served as the conformation-dependent distance.

As can be seen from Table S4, the reference distance is independent of conformation and varies within ±0.05 Å. The conformation-dependent distance in the conformer groups S and P is 3.54 ± 0.11 and 2.59 ± 0.08 Å, respectively. Integral NOESY cross-peak intensities as the functions of the mixing times were plotted (Figures S8 and S9); cross-relaxation rates were found to be 2.48 ± 0.09 × 10^–2^ and 1.10 ± 0.05 × 10^–2^ s^–1^ and 1.41 ± 0.06 × 10^–2^; 1.30 ± 0.08 × 10^–2^ s^–1^ for the distances H7–H10 and H22–H25/H29 inCDCl_3_ and DMSO-d_6_, respectively. In the slope of the ISPA model [31,32], the experimental effective distance was determined: 3.33 ± 0.05 Å for the studied system in CDCl_3_ and 2.94 ± 0.05 Å in DMSO-d_6_. By comparison of this number found from the NOESY experiments with those given by X-ray analysis, the populations of the umifenovir conformer groups(*x_s_*) in CDCl_3_ and DMSO were calculated using Equation (3):

(3) $$\frac{1}{r_{\exp}^{6}}=\frac{x_{s}}{r_{s}^{6}}+\frac{1-x_{s}}{r_{p}^{6}}\to x_{s}=\frac{r_{s}^{6}(r_{p}^{6}-r_{\exp}^{6})}{r_{\exp}^{6}(r_{p}^{6}-r_{s}^{6})}$$

where *r_s_* and *r_p_* are the interatomic distances in the conformer groups S and P, and *r_s_* is the effective distance derived from the NOESY data.

**Figure 3:**
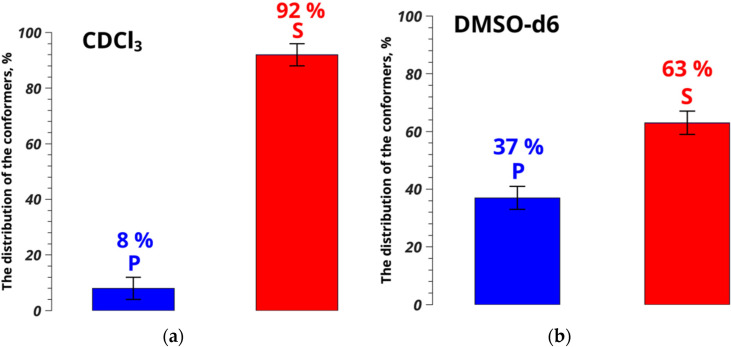
Conformer populations of umifenovir in CDCl_3_ (**a**) and DMSO-d_6_ (**b**), determined from NOESY data.

Thus, the distance between atoms H22 and H25/H29, characterizing changes in the umifenovir conformers, is 3.33 ± 0.05 Å in chloroform, and is similar to the value of 3.54 ± 0.12 Å found for solvates of umifenovir determined by XRD [27]. The difference of 0.21 Å is within the experimental accuracy level. At the same time, the polymorphic form consisting of conformers P has the distance H22–H25/H29 of 2.59 ± 0.08 Å. This differs from the value found from the NOESY experiments performed in chloroform by 0.74 Å, which is four times the experimental error. The results show that conformers S dominate over P as 92% to 8% for CDCl_3_, while in DMSO the corresponding populations ratio is 63% to 37% (Figure 3). Thus, formation of solvates of umifenovir in DMSO is unlikely, whereas chloroform is the preferred medium for screening of the solvate forms.

The methods described in this paper can provide a “finger-print” for predicting the probability of screening drug solvate crystal forms of umifenovir with certain molecular conformation.

## 4. Conclusions

It was found that the dominant conformation of umifenovir molecules dissolved in CDCl_3_ corresponds to the conformation appearing in stable crystal solvates, which is corroborated by the XRD data. At the same time, no prominent conformers were found in DMSO that would give the evidence of possible formation of solvates. The combined approach, based on NOESY and X-ray analysis data, turned out to be rather effective when solving structural problems related to APIs existing as multiple conformation-dependent, pseudo-polymorphic forms (e.g., crystal solvates). Results obtained in this work seem promising and can lead to further wide studies of APIs with pseudo-polymorphism, which is undoubtedly a necessary step in synthesis of new, and reprofiling of, existing pharmaceuticals.

## Data Availability

Not applicable.

## Supplementary Materials

The following supporting information can be downloaded at: https://www.mdpi.com/article/10.3390/pharmaceutics15010226/s1, Figure S1: ^1^H NMR spectrum of umifenovir (Arbidol) in CDCl3; Figure S2: ^13^C NMR spectrum of umifenovir in CDCl3; Figure S3: ^1^H-^13^C HSQC spectrum of umifenovir in CDCl3; Figure S4: ^1^H-^13^C HMBC NMR spectrum of umifenovir in CDCl3; Figure S5: ^1^H-^1^H TOCSY NMR spectrum of umifenovir in CDCl3 (mixing time 20 ms); Figure S6: ^1^H-^1^H TOCSY NMR spectrum of umifenovir in CDCl3 (mixing time 60 ms); Figure S7: ^1^H-^1^H TOCSY NMR spectrum of umifenovir in CDCl3 (mixing time 100 ms); Figure S8: Dependence of the average cross-peak intensity on the mixing time for the H7–H10 distance, obtained by analysis of the NOESY spectra of umifenovir in CDCl3; Figure S9: Dependence of the average cross-peak intensity on the mixing time for the H22–H25/29 distance, obtained by analysis of the NOESY spectra of umifenovir in CDCl3; Table S1: ^1^H and ^13^C chemical shifts and observed cross-correlation peaks in 2D spectra of umifenovir in CDCl3; Table S2: Spatial structure of the conformers of umifenovir obtained by CCDC; Table S3: Values of the dihedral angle τ_1_ of the umifenovir molecule in the considered conformers; Table S4: Conformation-dependent and reference distances in the umifenovir molecule.
