# Supplementary material for: Conformational Screening of Arbidol Solvates: Investigation via 2D NOESY
Source: Pharmaceutics. 2023 Jan 9;15(1):226. doi: 10.3390/pharmaceutics15010226 (PMC9865235; doi:10.3390/pharmaceutics15010226)
Supplement: Supplementary file 1 [file pharmaceutics-15-00226-s001.zip › pharmaceutics-2067357-supplementary.pdf]

pharmaceutics

Supplementary information

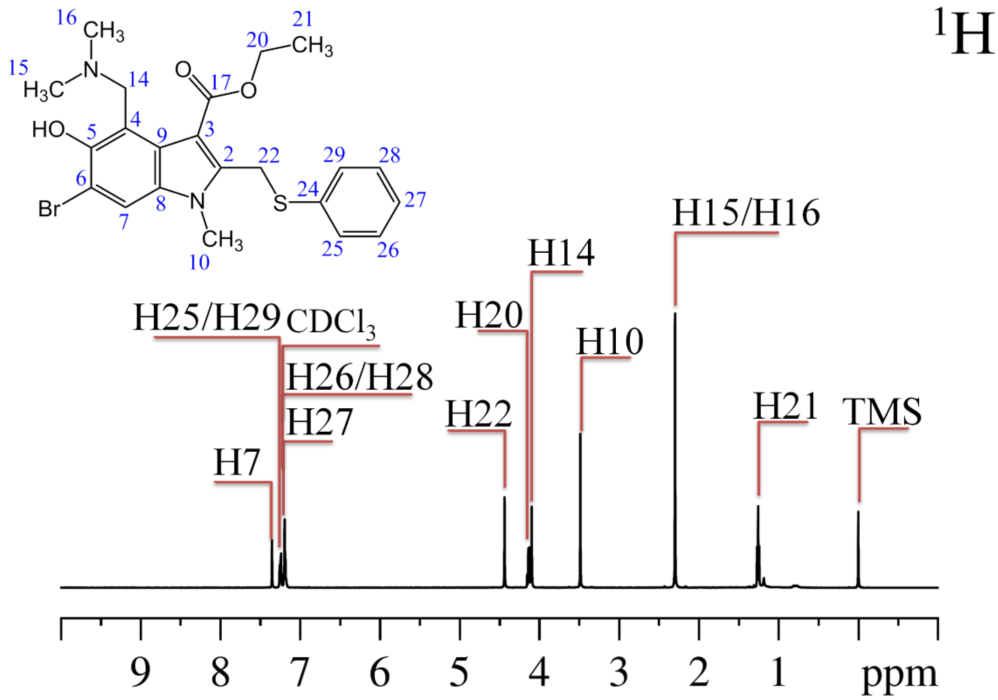

Figure S1. ^{1}H NMR spectrum of umifenovir (Arbidol) in $CDCl_3$.

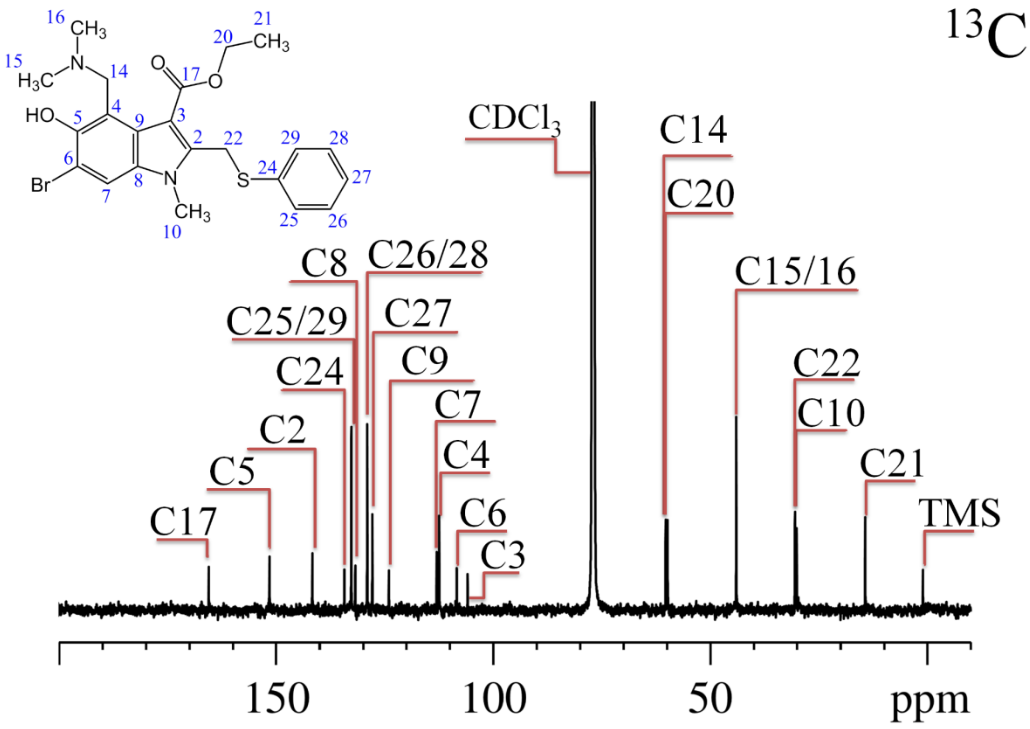

Figure S2. ^{13}C NMR spectrum of umifenovir in $CDCl_3$.

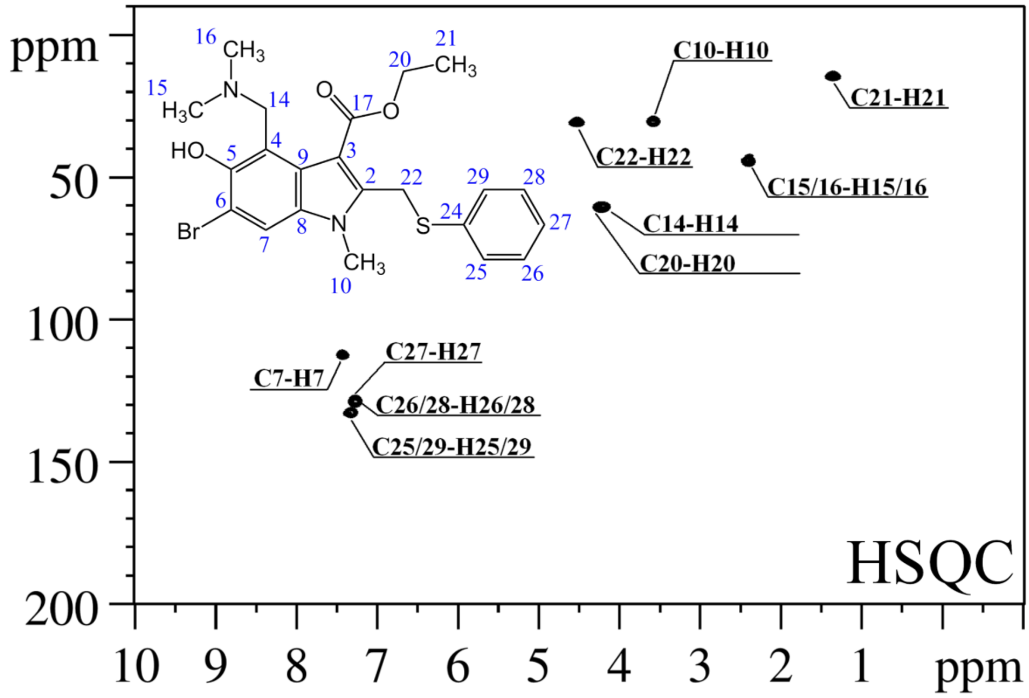

Figure S3. ^{1}H-^{13}C HSQC spectrum of umifenovir in $CDCl_3$.

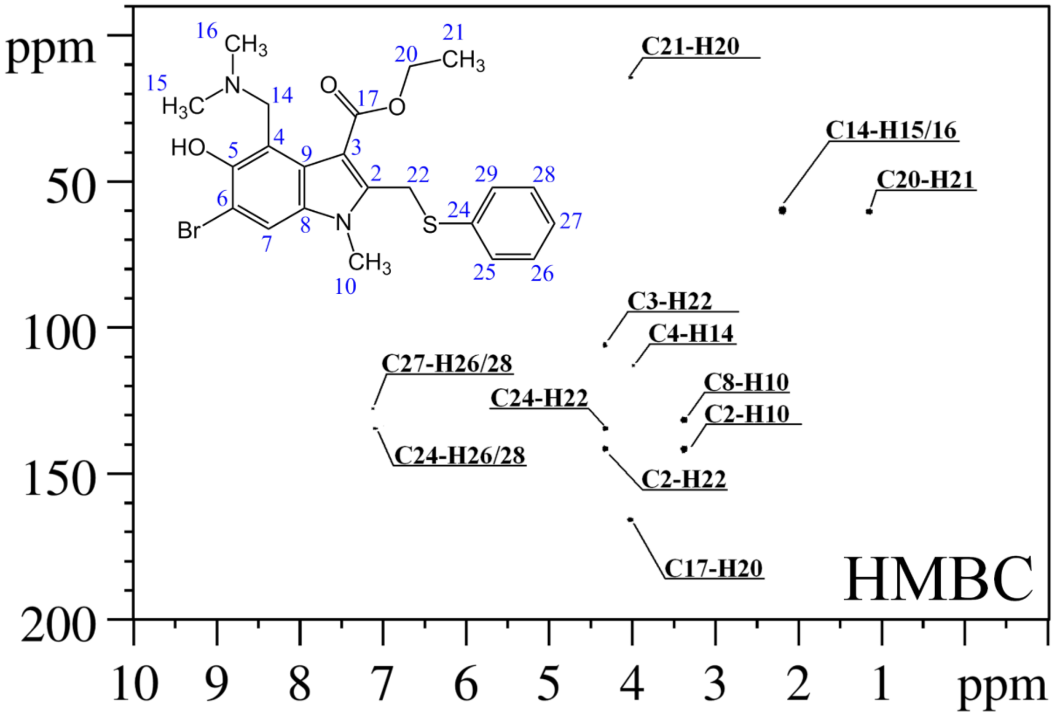

Figure S4. ^{1}H-^{13}C HMBC NMR spectrum of umifenovir in $CDCl_3$.

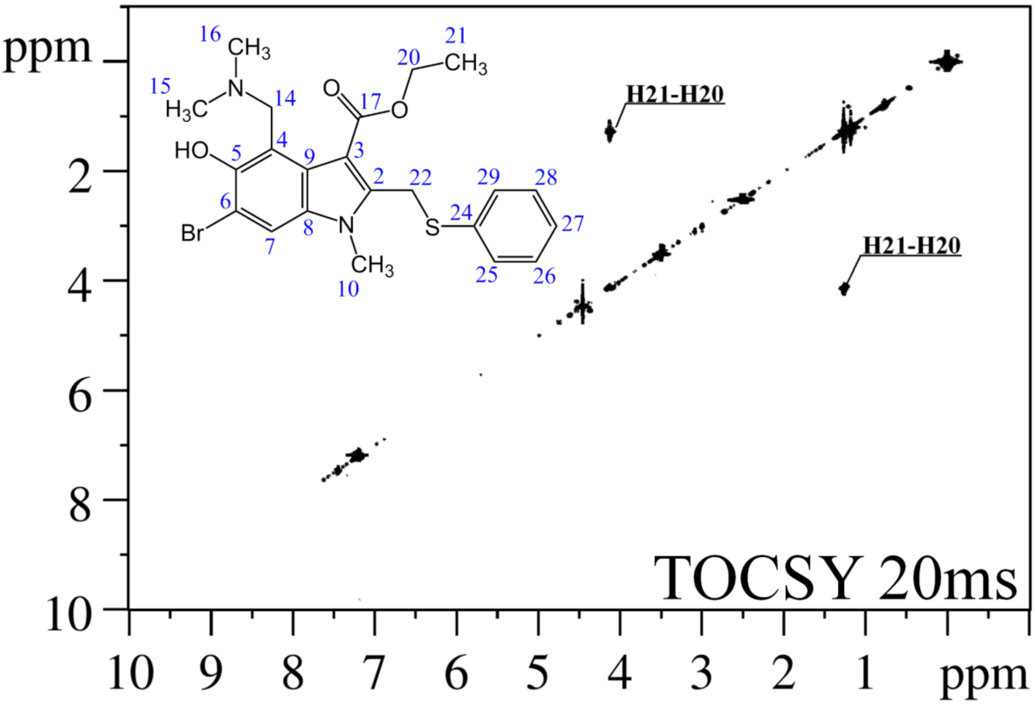

Figure S5. ^1H-^1H TOCSY NMR spectrum of umifenovir in $CDCl_3$ (mixing time 20 ms).

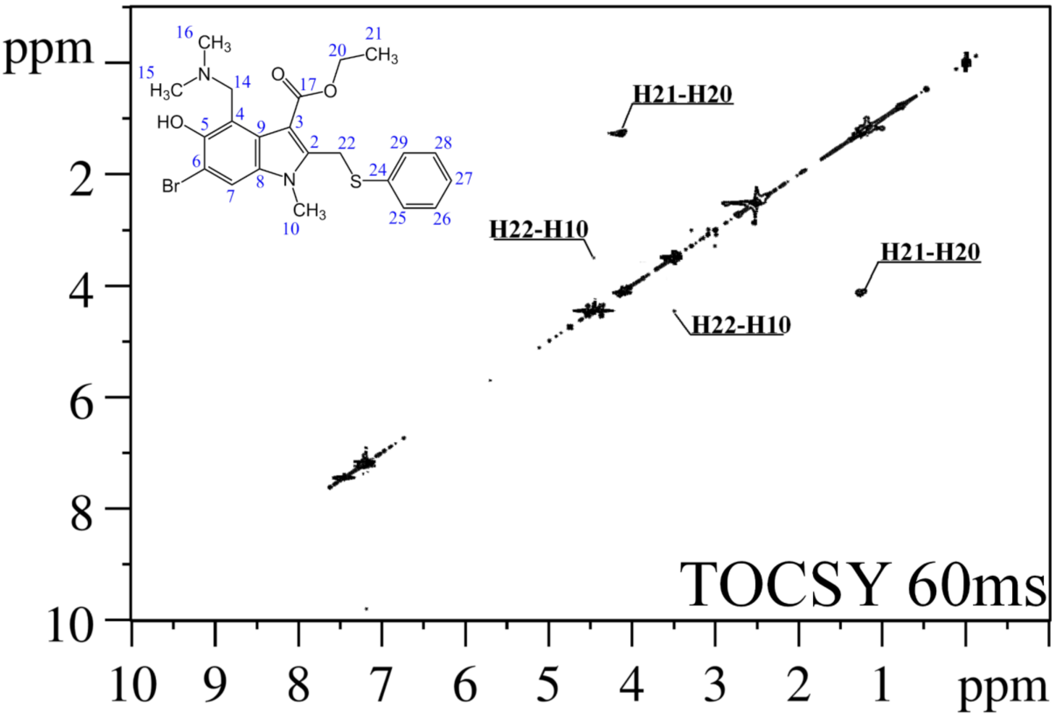

Figure S6. ^1H-^1H TOCSY NMR spectrum of umifenovir in $CDCl_3$ (mixing time 60 ms).

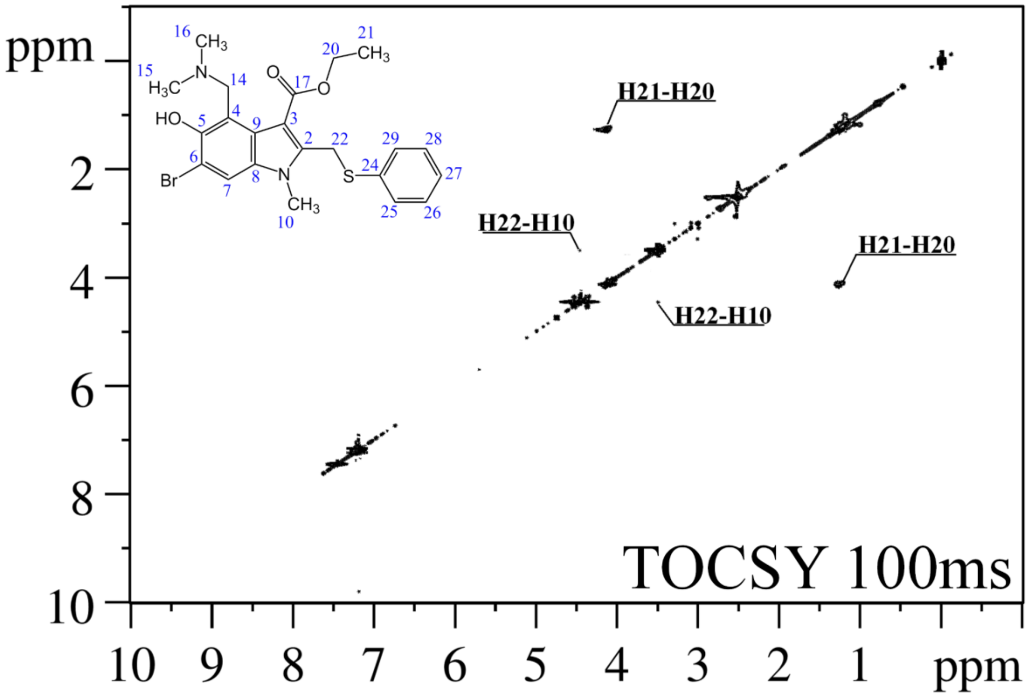

Figure S7. ^1H-^1H TOCSY NMR spectrum of umifenovir in $CDCl_3$ (mixing time 100 ms).

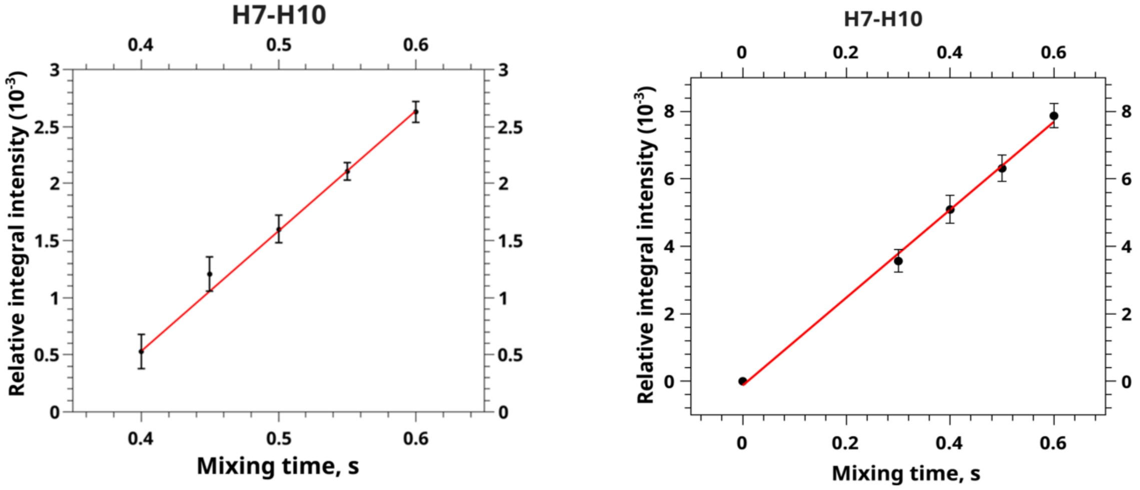

a b

Figure S8. Dependence of the average cross-peak intensity on the mixing time for the H7–H10 distance, obtained by analysis of the NOESY spectra of umifenovir in $CDCl_3$.

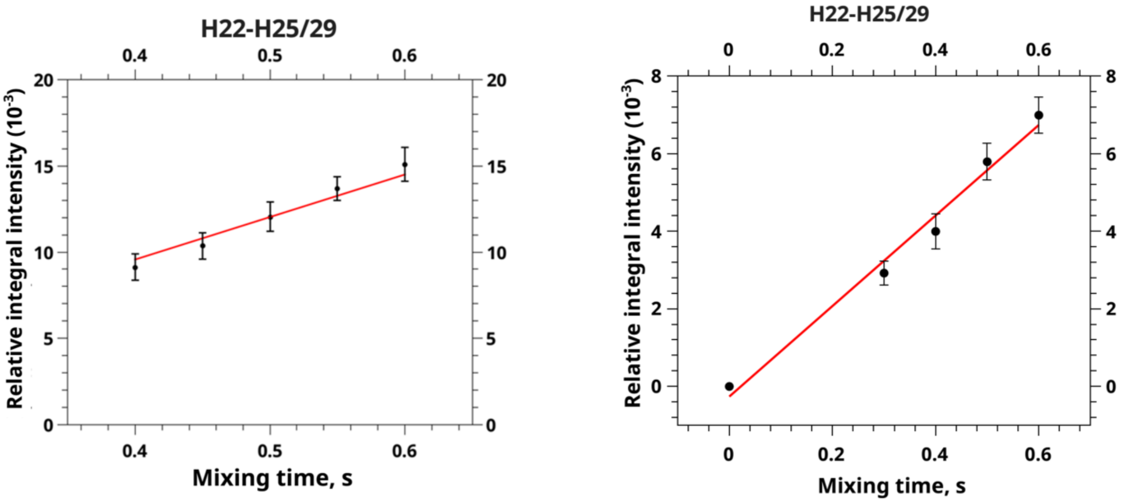

a b

Figure S9. Dependence of the average cross-peak intensity on the mixing time for the H22–H25/29 distance, obtained by analysis of the NOESY spectra of umifenovir in $CDCl_3$.

Table S1. ^{1}H and ^{13}C chemical shifts and observed cross-correlation peaks in 2D spectra of umifenovir in $CDCl_3$.

δ ^{13}C	^{1}H	δ ^{1}H	HMBC	HSQC	TOCSY			NOESY	^{13}C
					20ms	60ms	100ms		
141.20	-	-	C2-H10<br>C2-H22	-	-	-	-	-	C2
106.21	-	-	C3-H22	-	-	-	-	-	C3
113.51	-	-	C4-H14	-	-	-	-	-	C4
152.25	OH	1.31		-	-	-	-	-	C5
108.76	-	-		-	-	-	-	-	C6
112.86	H7	7.35		C7-H7	-	-	-	H7-H10	C7
131.77	-	-	C8-H10	-	-	-	-	-	C8
124.41	-	-		-	-	-	-	-	C9
28.97	H10	3.49		C10-H10	-	-	-	H10-H22	C10
59.74	H14	4.10	C14-H15/16	C14-H14	-	-	-	H14-H15/H16	C14
43.37	H15/H16	2.30		C15/C16-H15-H16	-	-	-	-	C15/C16
165.09	-	-	C17-H20	-	-	-	-	-	C17
59.66	H20	4.13	C20-H21	C20-H20	-	-	-	H20-H21	C20
14.04	H21	1.26	C21-H20	C21-H21	-	H20-H21	H20-H21	-	C21
29.90	H22	4.44	-	C22-H22	H20-H21	H22-H10	H22-H10	H22-H25/H29	C22
134.92	-	-	C24-H22<br>C24-H26/28	-	-	-	-	-	C24
132.18	H25-H29	7.25		C25/C29-H25/H29	-	-	-	-	C25/C29
128.81	H26/H28	7.20		C26/C28-H26/H28	-	-	-	-	C26/C28
127.31	H27	7.19	C27-H26/28	C27-H27	-	-	-	-	C27

Table S2. Spatial structure of the conformers of umifenovir obtained by CCDC.

S	
A	B
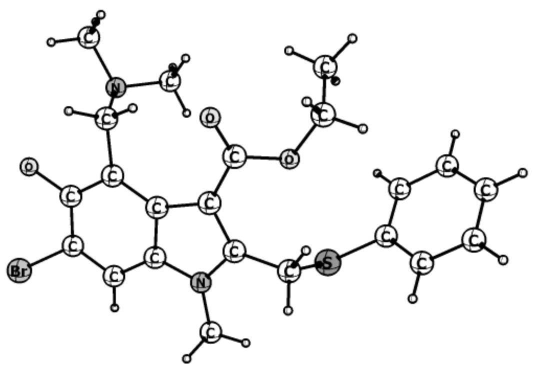	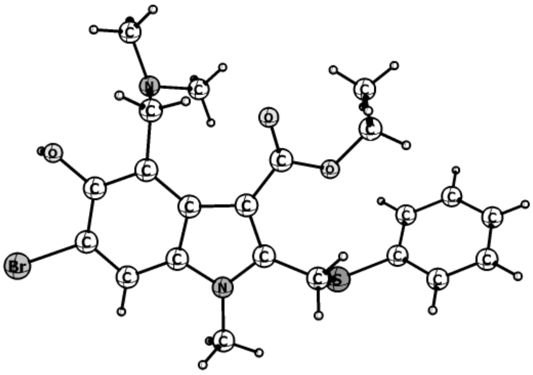
C	D
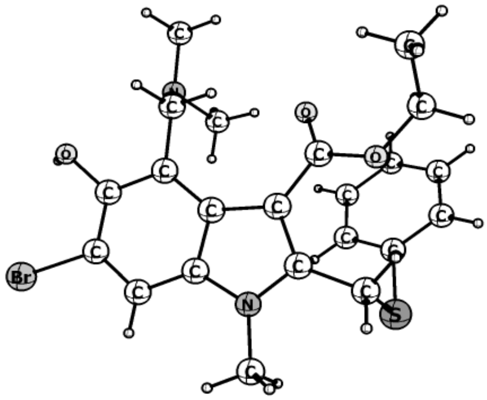	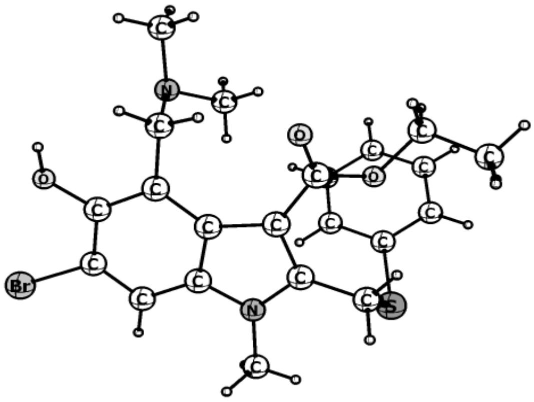
E	F
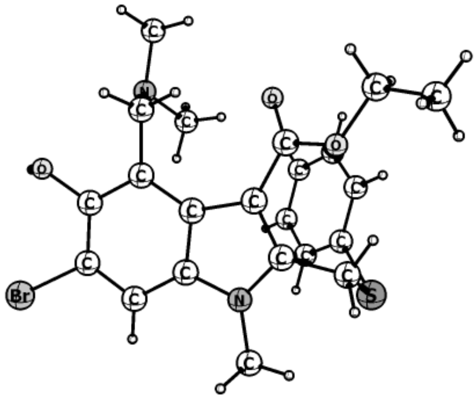	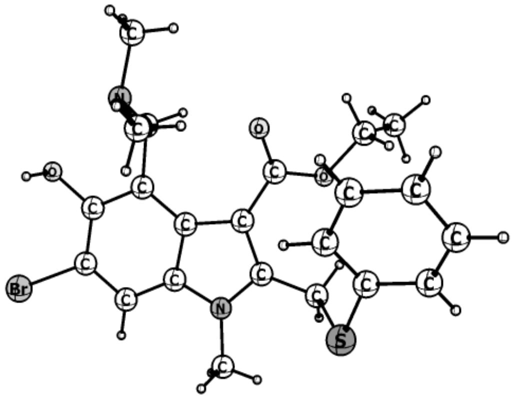
P	

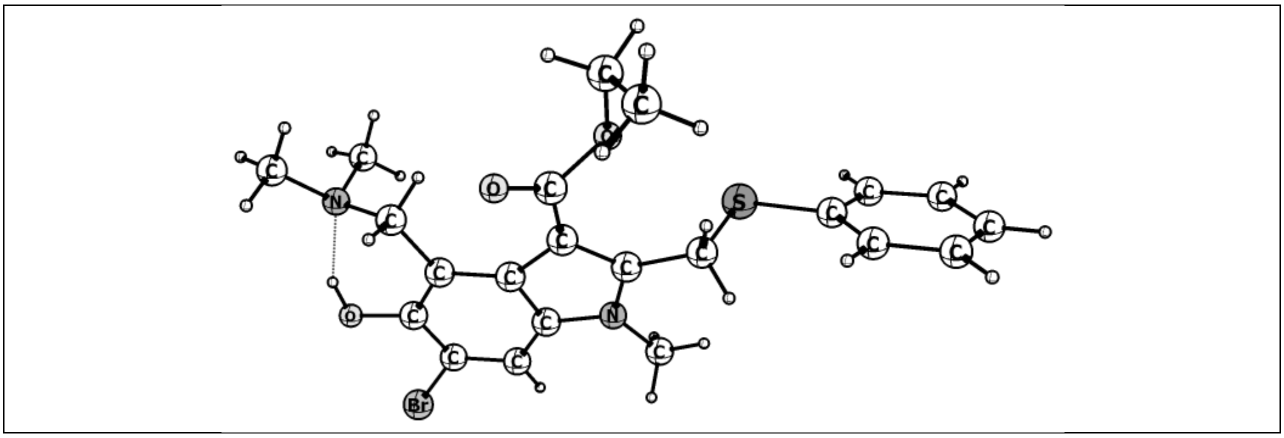

Table S3. Values of the dihedral angle τ_1 of the umifenovir molecule in the considered conformers.

Conformer	Values of the dihedral angle, °	
	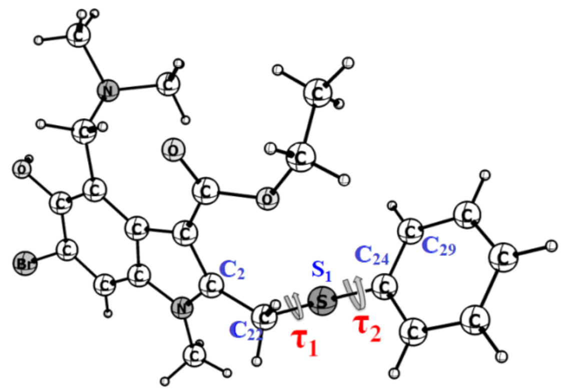	
	τ_1 (C_{22}-S_1-C_{24}-C_{29})	
S	A	–119.9
	B	–119.9
	C	–92.9
	D	–76.2
	E	–117.8
	F	67.8
P	169.2	

Table S4. Conformation-dependent and reference distances in the umifenovir molecule.

r (H7-H10), Å			r (H22-H25/29), Å		
Conf.		*r*, Å	Conf.		*r*, Å
S	A	2.94	S	A	3.21
	B	2.94		B	3.21
	C	2.78		C	3.72
	D	2.92		D	3.96
	E	2.75		E	3.36
	F	2.76		F	3.80
P		2.96	P		2.59
